# Supplementary material for: Human Nutrition Research in the Data Era: Results of 11 Reports on the Effects of a Multiple-Micronutrient-Intervention Study
Source: Nutrients. 2024 Jan 5;16(2):188. doi: 10.3390/nu16020188 (PMC10819666; doi:10.3390/nu16020188)
Supplement: Supplementary file 1 [file nutrients-16-00188-s001.zip › Kaput_Nutrients_File S1.pdf]

Figure S1

Flow Chart of Topics and Publications from Micronutrient Genomics Project

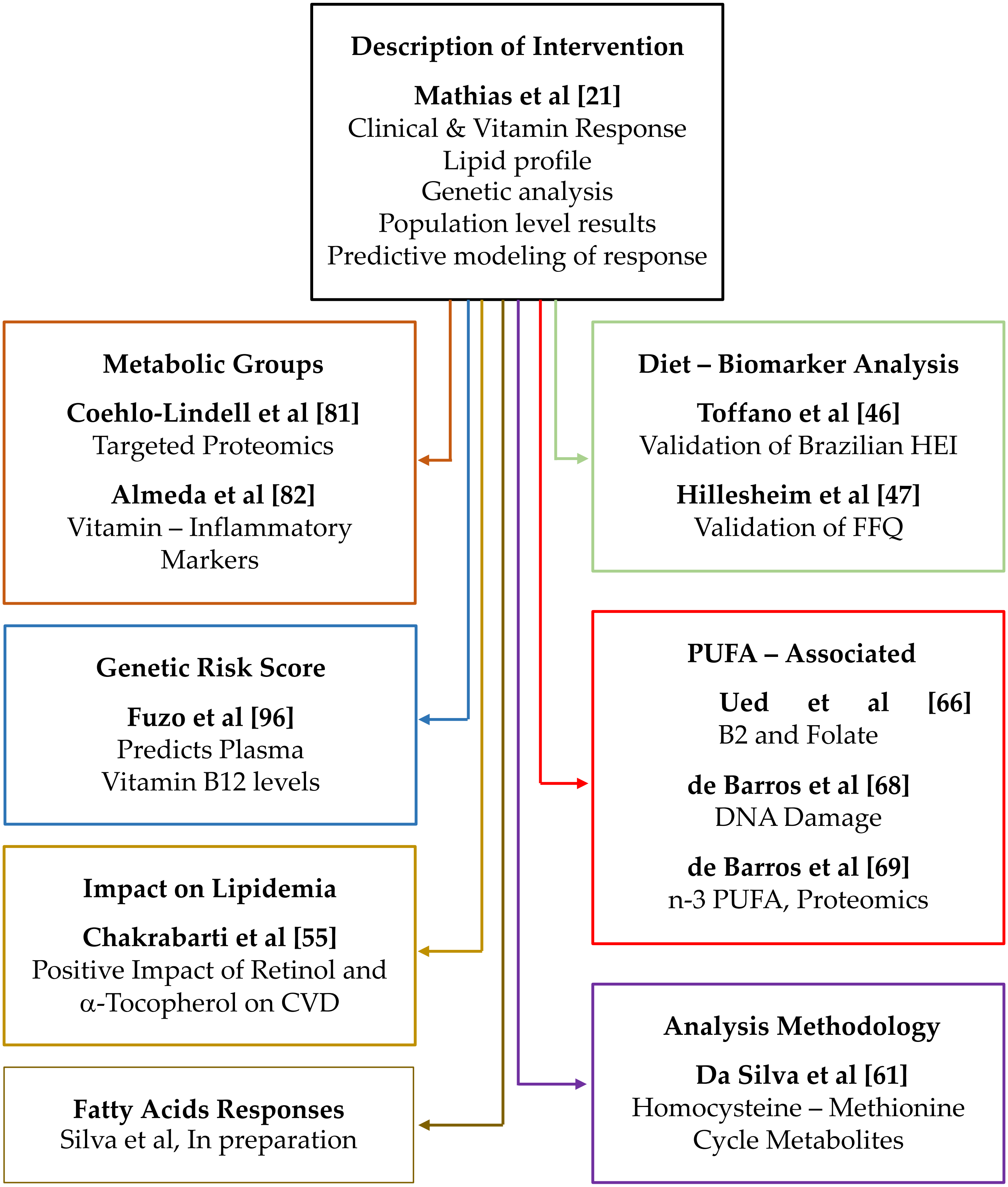

References

21. Mathias, M.G.M.G.; Coelho-Landell, C. d. A.C. de A.; Scott-Boyer, M.-P.M.P.; Lacroix, S.; Morine, M.J.M.J.; Salomão, R.G.R.G.; Toffano, R.B.D.R.B.D.; Almada, M.O.R. d. V.M.O.R. do V.; Camarneiro, J.M.J.M.; Hillesheim, E.; et al. Clinical and Vitamin Response to a Short-Term Multi-Micronutrient Intervention in Brazilian Children and Teens: From Population Data to Interindividual Responses. *Mol. Nutr. Food Res.* 2018, *62*, 1–73, doi:10.1002/mnfr.201700613.

46. Toffano, R.B.D.; Hillesheim, E.; Mathias, M.G.M.G.; Coelho-Landell, C.A.; Salomão, R.G.; Almada, M.O.R.V.; Camarneiro, J.M.; Barros, T.T.T.; Camelo-Junior, J.S.; Rezzi, S.; et al. Validation of the brazilian healthy eating in-dex-revised using biomarkers in children and adolescents. Nutrients 2018, 10, 1–14, doi:10.3390/nu10020154.

47. Hillesheim, E.; Toffano, R.B.D.; Barros, T.T. de; Salomão, R.G.; Mathias, M.G.; Coelho-Landell, C. de A.; Al-mada, M.O.R. do V.; Camarneiro, J.M.; Camelo-Junior, J.S.; Ued, F. da V.; et al. Biomarker-based validity of a food frequency questionnaire estimating intake in Brazilian children and adolescents. Int. J. Food Sci. Nutr. 2020, 72, 1–12, doi:10.1080/09637486.2020.1786026.

55. Chakrabarti, A.; Eiden, M.; Morin-Rivron, D.; Christinat, N.; Monteiro, J.P.; Kaput, J.; Masoodi, M. Impact of multi-micronutrient supplementation on lipidemia of children and adolescents. Clin. Nutr. 2020, 39, 2211–2219, doi:10.1016/j.clnu.2019.09.010.

66. Ued, F. V.; Mathias, M.G.; Toffano, R.B.D.; Barros, T.T.; Almada, M.O.R.V.; Salomão, R.G.; Coelho-Landell, C.A.; Hillesheim, E.; Camarneiro, J.M.; Camelo-Junior, J.S.; et al. Vitamin B2 and folate concentrations are associated with ARA, EPA and DHA fatty acids in red blood cells of Brazilian children and adolescents. Nutrients 2019, 11, 1–17, doi:10.3390/nu11122918.

61. Da Silva, L.; Collino, S.; Cominetti, O.; Martin, F.-P.F.-P.; Montoliu, I.; Moreno, S.O.S.O.S.O.; Corthesy, J.; Kaput, J.; Kussmann1, M.; Monteiro, J.P.J.P.; et al. High-throughput method for the quantitation of metabolites and co-factors from homocysteine – methionine cycle for nutritional status assessment. Bioanalysis 2016, 8, 1937–1949, doi:10.4155/bio-2016-0112.

68. de Barros, T.T.; Venâncio, V.D.P.; Hernandes, L.C.; Greggi Antunes, L.M.; Hillesheim, E.; Salomão, R.G.; Mathias, M.G.; Coelho-Landell, C.A.; Toffano, R.B.D.; Do Vale Almada, M.O.R.; et al. DNA damage is inversely associated to blood levels of DHA and EPA fatty acids in Brazilian children and adolescents. Food Funct. 2020, 11, 5115–5121, doi:10.1039/c9fo02551k.

69. de Barros, T.T.; Venancio, V. de P.; Hernandes, L.C.; Antunes, L.M.G.; Hillesheim, E.; Salomão, R.G.;Mathias, M.G.; Coelho-Landell, C.A.; Toffano, R.B.D.; Almada, M.O.R.D.V.; et al. Dna damage, n-3 long-chain pufa levels and proteomic profile in brazilian children and adolescents. Nutrients 2021, 13, 1–12, doi:10.3390/nu13082483.

81. Coelho-Landell, C.A.; Salomão, R.G.; Almada, M.O.R. do V.; Mathias, M.G.; Toffano, R.B.D.; Hillesheim,E.; Barros, T.T.; Camarneiro, J.M.; Camelo-Junior, J.S.; Rosa, J.C.; et al. Metabo groups in response to micronutrient in-tervention: Pilot study. Food Sci. Nutr. 2020, 8, 683–693, doi:10.1002/fsn3.1357.

82. Almada, M.O.R.D.V.; Almeida, A.C.F.; Ued, F. da V.; Mathias, M.G.; Coelho-Landell, C. de A.; Salomão R.G.; Toffano, R.B.D.; Camarneiro, J.M.; Hillesheim, E.; de Barros, T.T.; et al. Metabolic groups related to blood vit-amin levels and inflammatory biomarkers in Brazilian children and adolescents. J. Nutr. Sci. Vitaminol. (Tokyo). 2020, 66, 515–525, doi:10.3177/jnsv.66.515.

96. Fuzo, C.A.; da Veiga Ued, F.; Moco, S.; Cominetti, O.; Métairon, S.; Pruvost, S.; Charpagne, A.; Carayol, J.; Torrieri, R.; Silva, W.A.; et al. Contribution of genetic ancestry and polygenic risk score in meeting vitamin B12 needs in healthy Brazilian children and adolescents. Sci. Rep. 2021, 11, 1–15, doi:10.1038/s41598-021-91530-7.
