# Supplementary material for: Human Nutrition Research in the Data Era: Results of 11 Reports on the Effects of a Multiple-Micronutrient-Intervention Study
Source: Nutrients. 2024 Jan 5;16(2):188. doi: 10.3390/nu16020188 (PMC10819666; doi:10.3390/nu16020188)
Supplement: Supplementary file 1 [file nutrients-16-00188-s001.zip › Kaput_Nutrients_File S2.pdf]

File S2: Micronutrient Program Publications

McCabe-Sellers et al - 2008
Community Based
Participatory Research

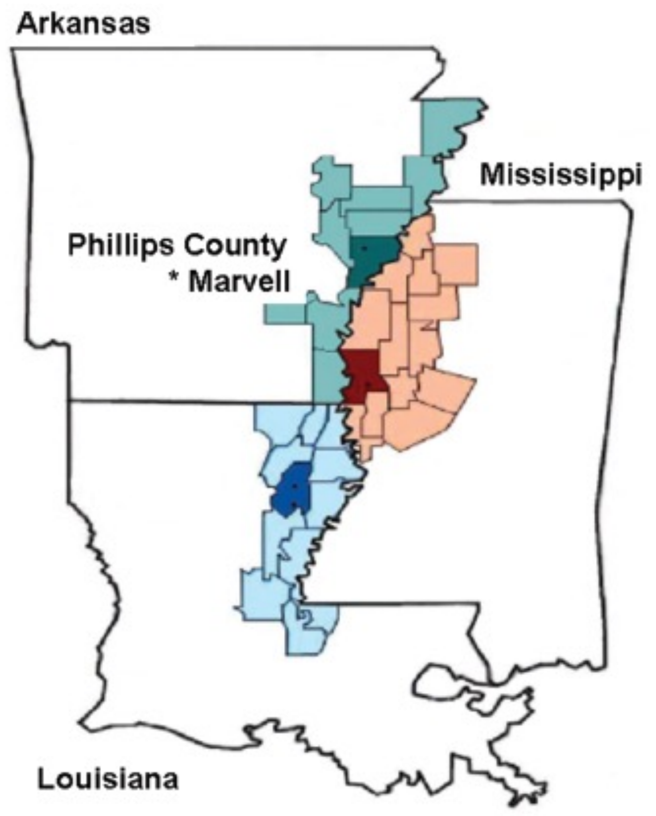

FIG. 1. The map and participants of the delta nutrition intervention research initiative. Shaded areas are the counties involved in the program, and dark shades are the "hubs" of each local.

Morine, Monteiro et al - 2014
Vitamin B2 and B9 association
with ARA, EPA, DHA

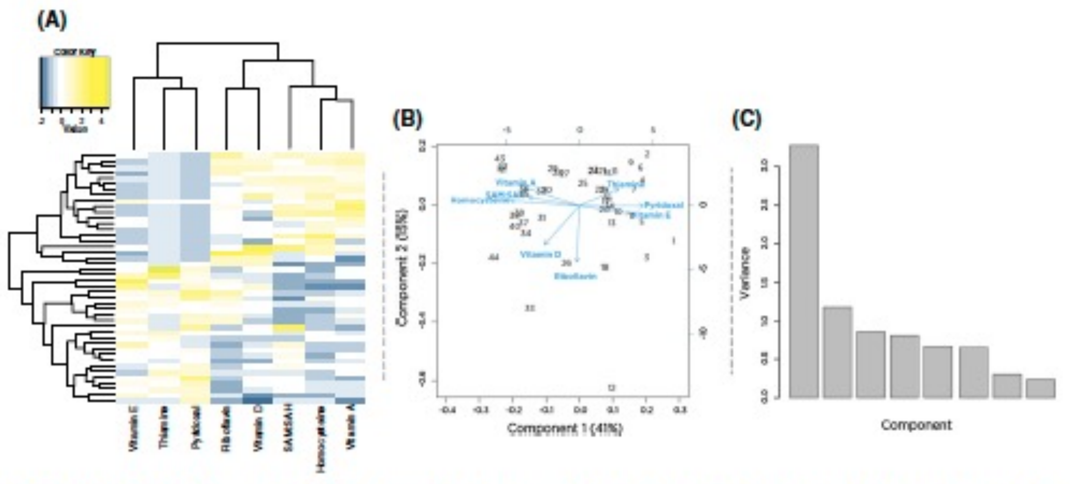

Fig. 1 Metabolite-level heat map and principal component analysis of vitamin levels. **a** Metabolite heat map where individuals are represented in the *rows*, and mean value of metabolite levels from three blood samplings is in the *columns*. **b** Principal component analysis of mean values of vitamin or metabolites. *Numbers* indicate values for individuals (**c**). Variances in each principal component (see "Materials and methods" section for details)

Scott-Boyer et al - 2016
Co-factor Network

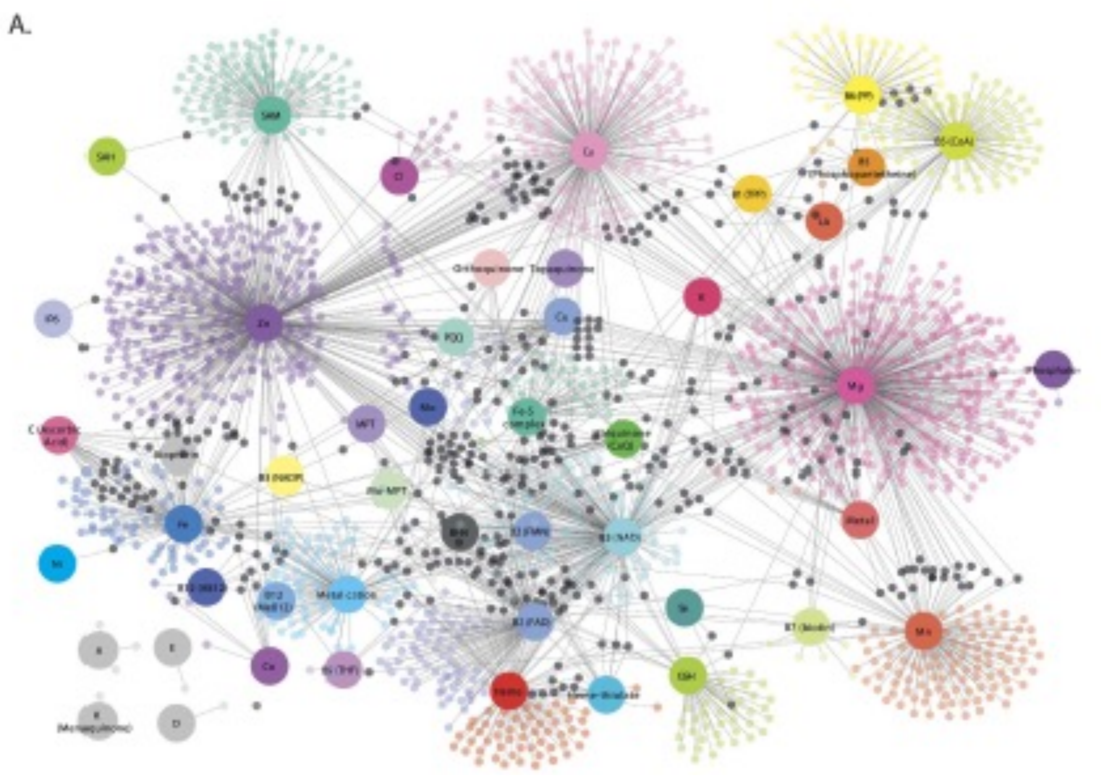

Mitchell et al - 2014
B Vitamins and Cognitive Function

Table 1
Genetic polymorphisms in B vitamin-related enzymes.

Gene	Enzyme function	Mutation effect	Disease association
Folate hydrolase (FOLH1) C484T, C1561T	Catalyzes the hydrolysis of N-acetylaspartylglutamate to glutamate and N-acetylaspartate	Unknown	Depression, schizophrenia (Roffman et al., 2013), dementia (Kim et al., 2010)
Methylene tetrahydrofolate reductase (MTHFR) C667T	Converts CH_3 THF to CH_2 THF	T homozygote is less efficient, thus increased plasma homocysteine	Depression, schizophrenia, mental retardation, dementia, bipolar disorder
Methionine synthase (MTR) A2756G	Converts homocysteine into methionine	G allele may increase homocysteine levels	Dementia, depression
Fucosyltransferase 2 (FUT2) (rs492602)	Immune response protein which modulates B12 transport in the gut	GG carriers have higher plasma B12	Intelligence
Dihydrofolate reductase (DHFR) 19bp deletion in the intron1 (rs70991108)	Converts dihydrofolate into tetrahydrofolate, using NADPH (for purine synthesis)	Reduces protein expression by eliminating Sp1 transcription factor binding site	Intellectual ability
Methylenetetrahydrofolate dehydrogenase (MTHFD1) G1958A	Converts 5,10-methylenetetrahydrofolate and $NADP^+$ into 5,10-methenyltetrahydrofolate and NADPH	A allele increases plasma homocysteine	Dementia
Cystathionine β synthase (CβS) 844ins68	Converts serine and homocysteine (with B6) into cystathionine	Insert increases plasma homocysteine	Dementia, schizophrenia
Methionine synthase reductase (MTRR or MSR) A66G	Converts SAH into SAM (with B12)	G allele increases plasma homocysteine	Mental retardation
Haptocorrin (TCN1) TC C776G	Protects cobalamin from degration in the stomach	Unknown	Dementia
Transcobalamin II receptor (TCN2) G775C	Binds cobalamin in the portal circulation	More efficient vitamin B12 transport and binding mechanisms versus R allele homozygotes	Depression
Folate receptor 1 (FOLR1) G1816A and G1841A	Activated by folate to induce signaling cascade	Double mutation (1816A and 1841A) possibly increases homocysteine levels	Tendency of double mutation (1816A and 1841A) to coincide with dementia

Kaput et al - 2014
Consensus Statement on
Micronutrient Research

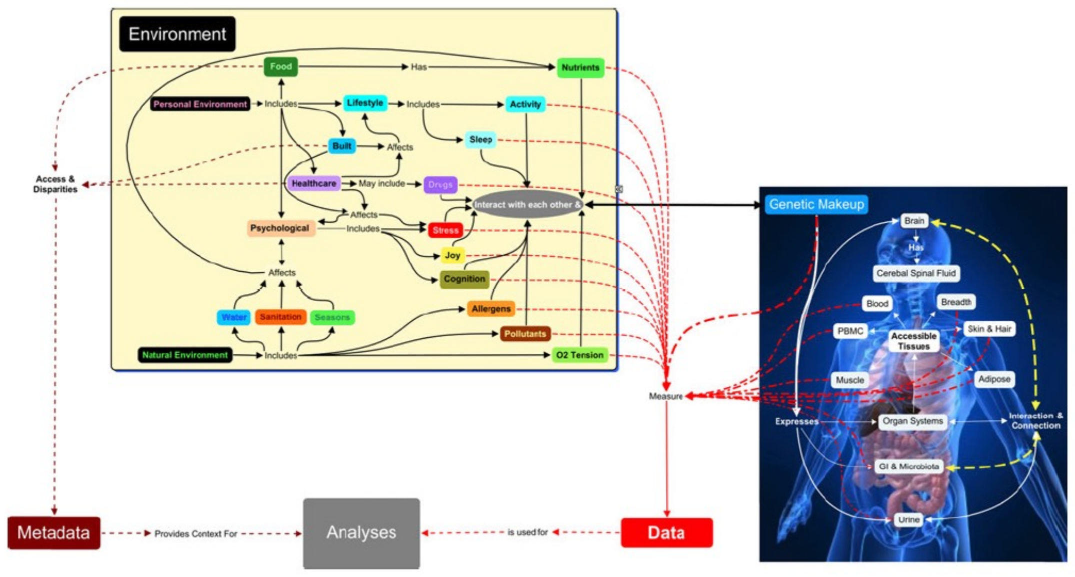

Parolo et al - 2017
Positive Selection of
Micronutrient Transporters

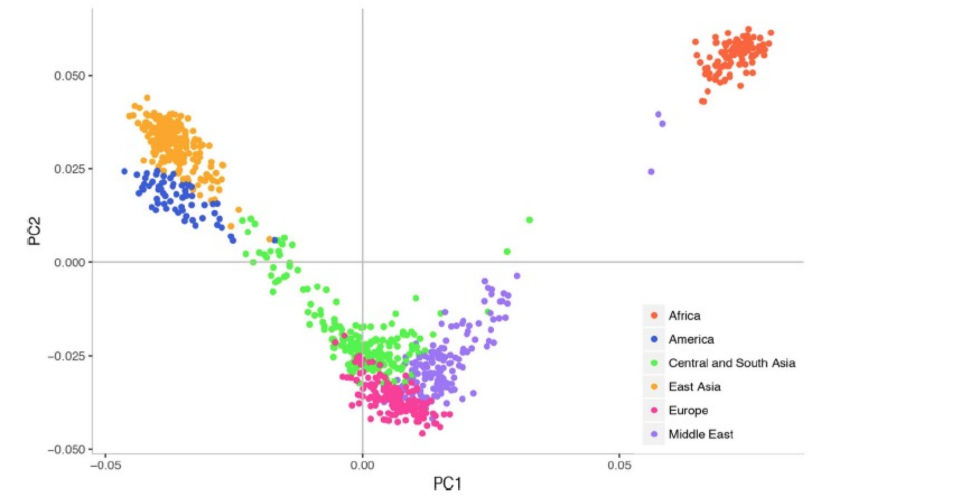

Fig. 1 PCA result. The scatterplot shows the first two components of PCA analysis based on genotype data of SNPs located in genes coding for transporters of cofactors in individuals from HGDP dataset. Each point corresponds to one individual, color-coded according to the geographic region of origin as shown in the legend

Monteiro et al - 2014
Methylation Potential & Diet, Genotype,
Protein, Metabolite Levels

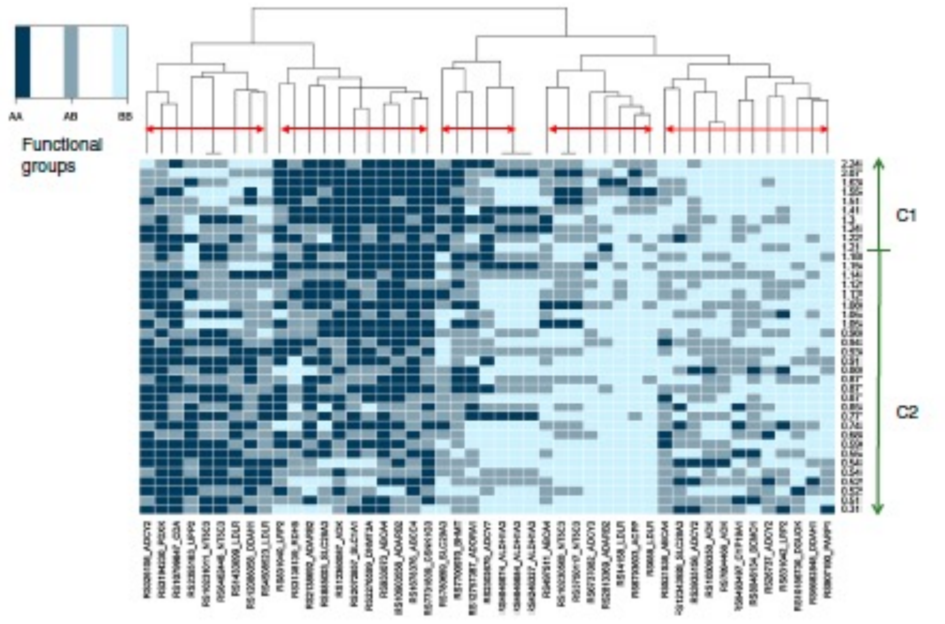

Fig. 2 Heatmap of significant SNPs associated with SAM/SAH ratios. SNPs statistically associated with SAM/SAH ratios (left axis, displayed high SAM/SAH to low) corrected for multiple comparisons were identified using procedures described in "Methods." Two-hundred and sixty-seven (267) genes were used for genetic analysis (Supplements 3 and 5)

Monteiro et al - 2015
Review

The genomics of micronutrient requirements

Hoeller et al - 2018
Review of Micronutrient Analysis

Table 1
Markers and methods for status determination of water-soluble vitamins; for details cf. the respective section in the text.

	Vitamin B_1	Vitamin B_2	Vitamin B_3 (Niacin)	Vitamin B_5 (Pantothenic acid)	Vitamin B_6	Vitamin B_7 (Biotin)	Vitamin B_9 (Folate)	Vitamin B_{12}	Vitamin C
Best marker	Erythrocyte t ransketolase, erythrocyte thiamine diphosphate	Erythrocyte glutathione reductase	Plasma niacin metabolites, but not reliable	Pantothenic acid after liberation of bound forms	Pyridoxal-5″-phosphate	Urinary biotin	Serum folate (short-term status) and red cell folate (long-term status)	Serum total cobalamin gradually being replaced by holoTC Confirmatory status marker methyl malonic acid	Serum ascorbate
State of the art methodology	Erythrocyte transketolase activity coefficient (ETKac) assay or HPLC analysis of whole blood or erythrocytes	EGRac assay or LC-MS/MS analysis of riboflavin and FMN	LC-MS/MS	LC-MS/MS	HPLC or LC-MS/MS	LC-MS/MS or microbiology	LC-MS/MS and newer microbiological methods.	GC-MS for MMA; binding assays linked to fluorescence detection systems for B_{12} and holoTC.	HPLC
Matrix[a]	Washed red blood cells	Washed red blood cells	Plasma, Urine	Whole blood	Plasma or serum	Urine	Serum and whole blood lysed into 1% ascorbic acid	Serum or plasma	Plasma or serum
Concentration range (matrix and marker)[b]	132-284 nmol/L (erythrocyte, thiamine diphosphate)	1.00–1.10 (erythrocyte, EGRac coefficient)		1.57–2.66 μmol/L (whole blood, pantothenic acid)	49.8 ± 1.2 nmol/L (plasma, pyridoxal-5″-phosphate)	6-50 μg/24-hr (urine, biotine)	13.4–44.5 nmol/L (serum, folate)	238 ± 102 pmol/L (plasma, vit. B_{12})	63.0 ± 19.9 μmol/L (plasma, ascorbic acid)
Gaps/issues	Lack of validated status cut-offs. Assay standardization against true biological function; inter-laboratory standardization required	Lack of validated status cut-offs. Assay standardization against true biological function; inter-laboratory standardization required	Validated plasma markers of niacin status	Simple sample preparation methodology	Inter-laboratory standardization required		Analysis of red cell folates by LC-MS/MS. Issues with deconjugation of folates to mono-glutamate forms. Affinity of binding protein assays to different folate derivatives.	Factors influencing MMA that are not related to B_{12} status	Inter-laboratory standardization required; results influenced by stabilization at time of collection
Outlook: promising techniques or development	In the field alternative; point-of-care analysis; LC-MS/MS analysis of free and phosphorylated thiamine forms	In the field alternative; point-of-care analysis	In the field alternative; point-of-care analysis		Dried blood spotting; point-of-care analysis; validation of metabolite ratios		LC-MS/MS. Analysis of Guthrie cards is in progress but validation against serum/red cell folate is required.	HoloTC assay	In the field alternative; point-of-care analysis

[a] All matrices are derived from venous blood, typically 100 μL is required for analysis.
[b] Please note that these data are only indicative of the physiological range in the specified matrix as reported in the literature[4] and that they may vary according to the population and used analytics. Consequently, they are not meant to be used as reference ranges nor to define micronutrient deficiency and nutritional recommendations.
