# Supplementary material for: Human Nutrition Research in the Data Era: Results of 11 Reports on the Effects of a Multiple-Micronutrient-Intervention Study
Source: Nutrients. 2024 Jan 5;16(2):188. doi: 10.3390/nu16020188 (PMC10819666; doi:10.3390/nu16020188)
Supplement: Supplementary file 1 [file nutrients-16-00188-s001.zip › Kaput_Nutrients_File S3.pdf]

File S3

Nestrovit Composition, DRI's, and UL [a]

**Micronutrient**	**Chemical Form**	**2 Tablets [b] (10g)**	**3 Tablets [c] (15g)**	**DRIs** 9 - 13 y	**UL** 9 – 13 y
Vitamin A	Retinol palmitate	534 µg	801 µg	600 µg	1700 µg
Vitamin E	α-Tocopherol acetate	6.6 mg	9.9 mg	11 mg	600 mg
Folate	Tetrahydrofolic acid	133.3 µg	200 µg	300 µg	600 µg
Vitamin B1	Thiamine pyrophosphate	0.93 mg	1,4 mg	0.9 mg	-
Vitamin B2	Flavin adenine dinucleotide	1.17 mg	1.76 mg	0.9 mg	-
Niacin	Nicotinamide diphosphate	12 mg	18 mg	12 mg	20 mg
Vitamin B6	Pyridoxal 5-phosphate	1.33 mg	2 mg	1.0 mg	60 mg
Vitamin B12	Methylcobalamin	0.73 µg	1.1 µg	1.8 µg	-
Vitamin D3	Cholecalciferol	3.4 µg	5.1 µg	5.0 µg	50 µg
Vitamin C	Calcium ascorbate	40 mg	60 mg	45 mg	1200 mg
Biotin	Biotin	13.3 µg	20.0 µg	20 µg	-
Vitamin B5	Pantothenic acid	4 mg	6 mg	4 mg	-
Calcium	Calcium	191.3mg	287 mg	1300 mg	2500 mg
Phosphorus	Phosphorus	144.6mg	217 mg	1250 mg	4000 mg
Iron	Iron	4,3 mg	6.5 mg	8 mg	40 mg
Magnesium	Magnesium	83.3 mg	125 mg	240 mg	350 mg
Zinc	Zinc	5.3 mg	6 mg	8 mg	23 mg

[a] Composition in 2013 and 2014. Newer versions differ in nutritional and vitamins content (https://www.nestrovit.ch)

[b] Two tablets provided 52 kilocalories (primarily from 0.52 g protein, 4.4 g carbohydrate, and 3.4 g of fat) which represents 3.3% of the recommended energy intake for a sedentary 9 to10 years old child (1600kcal/day).

[c] Three tablets provided 77 kilocalories (primarily from 0.8g protein, 6.7g carbohydrate, and 5.2g of fat) which represents 4.3% of the recommended energy intake for a sedentary 11 to 12 years old adolescent (1800kcal/day) and 3.9% of the recommended energy intake (2000 kcal/day) for a sedentary 13 years olds.

[d] The USDA Scientific Report of the 2015 Dietary Guidelines Advisory Committee (pages 101 – 102 of [90]) stated that boys and girls in the 9-13 years old group may be vulnerable to nutritional risks since they do not consume adequate amount of fruits and vegetables and their intake of refined grains is high.
