# Supplementary material for: Human Nutrition Research in the Data Era: Results of 11 Reports on the Effects of a Multiple-Micronutrient-Intervention Study
Source: Nutrients. 2024 Jan 5;16(2):188. doi: 10.3390/nu16020188 (PMC10819666; doi:10.3390/nu16020188)
Supplement: Supplementary file 1 [file nutrients-16-00188-s001.zip › Kaput_Nutrients_File S4.pdf]

File S4

Impact of multi-micronutrient supplementation on lipidemia of children and adolescents

Clin Nutr **39**, 2211-2219 (2019)

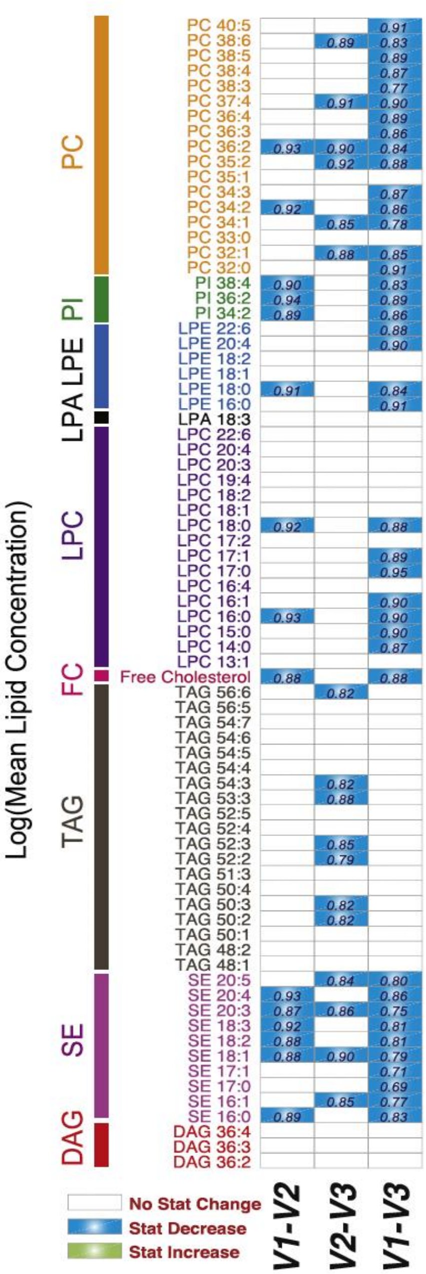

Fig. S2. Changes in lipid levels measured across visits. Increase or decrease in lipid levels in cross-visit comparisons are indicated by blue (decrease) or green (increase) filled boxes. Mean fold changes of lipids across visits are indicated by the numbers in the boxes.

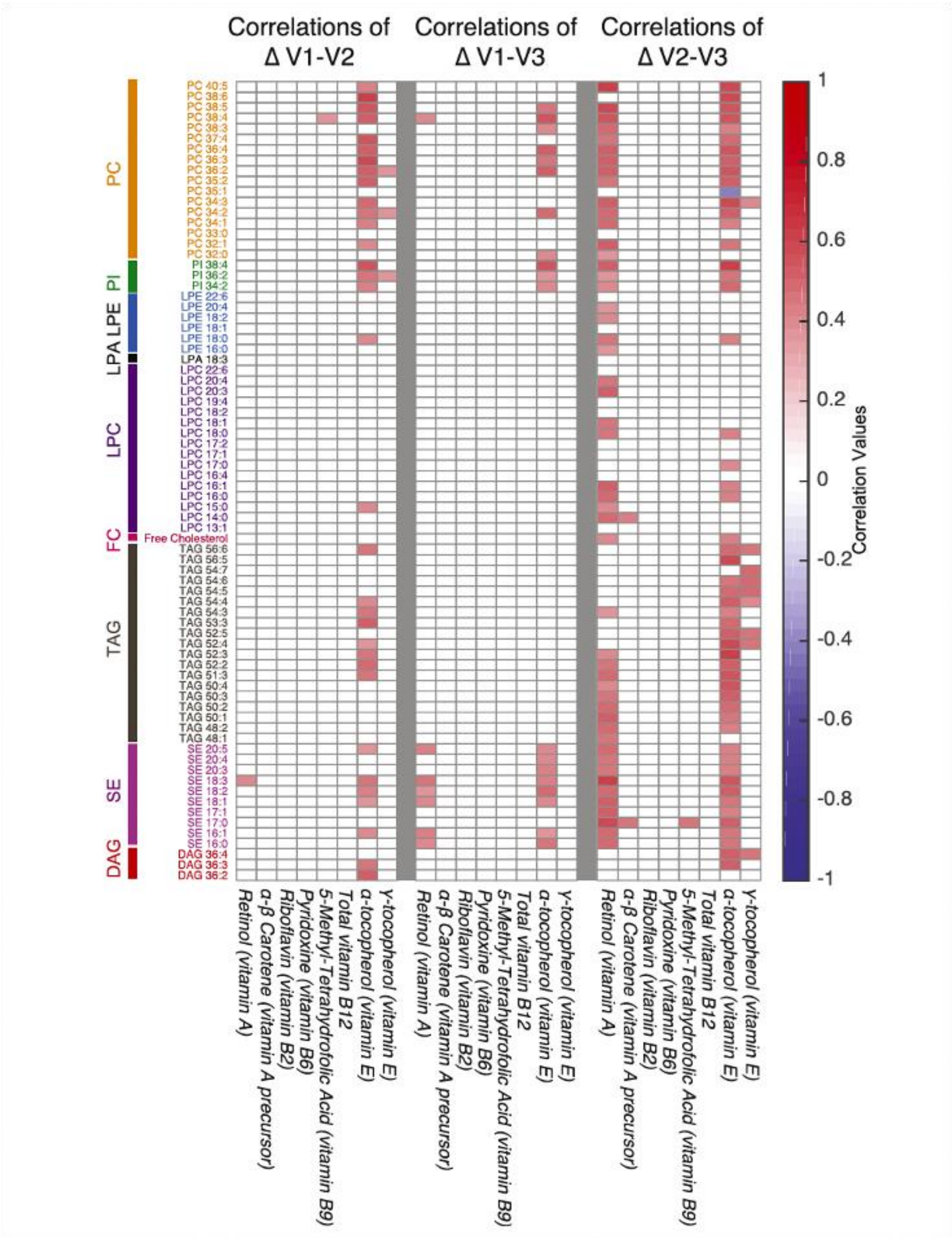

Fig. S3. Correlations of changes in measured lipid species (deltas between the visits) against changes in cholesterol measurements.
